# Early response activation in repetition priming: an LRP study

**DOI:** 10.1007/s00221-017-5017-1

**Published:** 2017-07-12

**Authors:** Christian Valt, Birgit Stürmer, Werner Sommer, Stephan Boehm

**Affiliations:** 1 Wales Institute for Cognitive Neuroscience, School of Psychology, Bangor University, Bangor, Gwynedd, Wales UK; 2 International Psychoanalytic University, Stromstr. 3b, 10555 Berlin, Germany; 3 Humboldt-Universität zu Berlin, Berlin, Germany

**Keywords:** Repetition priming, Motor activation, Lateralized readiness potential, Decision/action code retrieval, Rapid response learning

## Abstract

According to recent interpretations of repetition priming, response codes are automatically bound to a stimulus and retrieved during successive presentations of the stimulus, hence, affecting its current processing. Despite a solid corpus of behavioural evidence in line with this interpretation, electrophysiological studies have reported contrasting results regarding the nature and the timing of response code retrieval. The present experiment aims to establish at which stage of information processing decision and action codes are retrieved in repetition priming. To this end, the lateralized readiness potential (LRP) was analysed for primed faces to monitor motor cortex activity related to response preparation. Congruent and incongruent responses were obtained by having identical or reversed tasks between study and test. Primed stimuli presented LRP activations with opposite polarities for the two congruency conditions in the time-window 250–300 ms, indicating response-related motor cortex activity resulting from the retrieval of correct and incorrect decision/action codes for congruent and incongruent trials, respectively. This result indicates that decision and action codes bound to a primed stimulus are retrieved at early stages of stimulus processing and that these codes are transmitted to the motor cortex.

## Introduction

Any experience leaves a trace in the brain that affects future processing. In experimental psychology such adaptations can be seen in faster and more accurate performance to repeated stimuli (primed) compared to initial stimulus presentation (unprimed), so-called repetition priming (Richardson-Klavehn and Bjork 1988; Roediger and McDermott 1993).

Many theoretical accounts suggest that repetition priming results from facilitation within the perceptual and conceptual representational systems of a stimulus (Bruce and Young 1986, 2012; Burton 1998; Humphreys et al. 1995; Morton 1969; Moscovitch 1992; Richardson-Klavehn and Bjork 1988; Roediger and McDermott 1993; Squire 2004; Tulving and Schacter 1990). However, recent studies showed that priming could also arise from processes related to response selection and execution (Henson et al. 2014). According to the theory of rapid response learning (Dobbins et al. 2004), the response made to a stimulus is bound to the stimulus as instance (Logan 1990) or event file (Hommel 2004), and automatically retrieved when the stimulus recurs. Such retrieval allows by-passing some processing stages and facilitates response selection for primed compared to unprimed stimuli (Dobbins et al. 2004). Alternatively, the retrieved response may interact with the response obtained from stimulus processing and priming is determined by the congruency of the responses (Horner and Henson 2009).

Although facilitation in perceptual and conceptual networks and rapid response learning have been often presented as alternative theories of repetition priming, Valt et al. (2015) showed that these two theories are complementary. Repetition priming in the domain of person recognition is mainly determined by perceptual and conceptual facilitation and, under specific circumstances, such as multiple presentations at study, may be further supported by the retrieval of stimulus–response bindings. In effect, priming for repeated stimuli is reduced, but not abolished, when the response required at test is different from the response made at study, for example, after task reversal or task change. The presence of residual priming even after task modification is evidence of forms of priming resistant to response changes resulting from variations of the task, as predicted by facilitation in perceptual and conceptual networks (Boehm and Sommer 2012). On the other hand, reduction in priming after reversing the stimulus–response mapping is in line with automatic retrieval of a stimulus–response binding created at study that interacts with the new stimulus–response mapping in the test phase (Horner and Henson 2009).

The concept of stimulus–response binding is not new in the priming literature (Hommel 2004; Logan 1990). Effects of response congruency have been observed between subsequent stimuli, even when the preceding stimulus was a distracter (Rothermund et al. 2005) or was masked (Damian 2001), but these effects were short-lived and abolished by intervening stimuli (Frings 2011). Instead, stimulus–response bindings created by rapid response learning are robust and long-lasting (Dennis et al. 2010; Dennis and Perfect 2013; Dennis and Schmidt 2003; Dew and Giovanello 2010; Horner and Henson 2009, 2011, Schnyer et al. 2007; Schnyer et al. 2006; Soldan et al. 2012). Moreover, bindings created by rapid response learning include multiple stimulus levels (Horner and Henson 2011) and response codes (Dennis and Perfect 2013; Horner and Henson 2009): classification (e.g. “bigger” than a shoebox), decision (e.g. yes), and action (e.g. left button press). These bindings are influenced differently by task manipulations between study and test. Tasks requiring different types of semantic information impair the classification code, whereas question reversal within the same semantic task concurrently affects the decision and the action codes (Valt et al. 2015). The individual effects of these two codes can be isolated by changing the mapping of “yes” and “no” responses to the buttons (Dennis and Perfect 2013). Studies on rapid response learning have preferentially investigated the combined effect of decision and action codes (Dobbins et al. 2004; Horner and Henson 2009, 2011; Schnyer et al. 2007; Schnyer et al. 2006; Soldan et al. 2012; Valt et al. 2015), probably because the joint contribution of the decision and the action codes leads to highly robust effects (Dennis and Perfect 2013).

Bindings created by rapid response learning represent a complex type of long-term memory, whose understanding is important for memory models. However, despite some experimental attempts at clarification (Horner and Henson 2012; Hsu and Waszak 2012; Race et al. 2010), the time-course of response code retrieval and its electrophysiological correlates are still unclear. The present experiment addresses, based on the preference shown in prior studies, the combined effect of decision and action binding on the electrophysiological response evoked by primed stimuli.

Event-related potentials (ERPs) associated with the retrieval of response codes bound to a stimulus by rapid response learning have been investigated previously in three studies. Race et al. (2010) employed a design in which tasks were either reversed or changed between successive stimulus presentations and found an ERP correlate of decision/action code retrieval around 450 ms after stimulus onset. On the other hand, Horner and Henson (2012) found that after a change of the referent object in a size decision task the retrieval of all response codes (classification, decision, and action) affected ERPs time-locked to the response but not stimulus-locked ERPs (see also Race et al. 2010). Response-locked ERP waves for the two priming conditions differed between 200 and 100 ms before response. In a third study, Hsu and Waszak (2012) investigated the effect of changing response mappings within or between tasks on priming and did not find any ERP modulations related to action code retrieval. The diverging findings of these three ERP studies could result from the use of different experimental designs, and the consequent manipulation of different response codes.

The lateralized readiness potential (LRP) may be more specific than ERPs to analyse pure response processes, because it directly reflects motor cortex activity (Coles 1989) and is, therefore, a promising method to study the time-course of response code retrieval and its influence on response selection and execution within a repetition priming design. The LRP is calculated from recordings over the left and right hand area of the motor cortices in choice-response tasks involving manual responses. LRP deflections start after the response hand is selected and reflect hand-related motor activation (Coles 1989; Masaki et al. 2004). Due to the calculation method, negative LRP deflections reflect preparation of the correct response hand; positive deflections indicate preparation of the incorrect response hand.

The LRP has been often used to investigate response activation in cognitive conflict tasks like the Simon task (Simon 1969) or the Eriksen task (Eriksen and Eriksen 1974). Cognitive conflict effects are mainly accounted for by dual-route models of response selection (Kornblum et al. 1990). Here, response selection takes place via an indirect route accomplished by the task-relevant stimulus–response mapping. However, in cognitive conflict tasks, task-irrelevant stimulus features overlap with the response and automatically activate corresponding responses via a secondary direct route. In congruent trials, task-relevant and task-irrelevant stimulus features point towards the same response, hence do not provoke any conflicts. In incongruent trials, stimulus features point towards different responses resulting in conflicts that have to be solved before the correct response can be executed. LRPs in such incongruent trials show an early positive deflection (“dip”) indicating motor activation of the incorrect response hand, which is later replaced by a negative deflection when the correct response is selected via the indirect route (Gratton et al. 1988; Stürmer et al. 2002).

The idea that response codes are retrieved in repetition priming fits well with the dual-route framework. In effect, a dual-route interpretation of repetition priming has been suggested in a hybrid model (Horner and Henson 2009; Valt et al. 2015). According to this model, bindings between stimuli and response codes are created and, once established, automatically retrieved even if they are task-irrelevant, corresponding to the direct route in the dual route framework. Simultaneously, a response is also generated based on the stimulus processing in perceptual and conceptual networks by taking into account potential new task requirements, corresponding to the indirect route. These two responses—based on response bindings and stimulus re-processing—then interact, leading to facilitation when congruent and a reduction of facilitation, or even inhibition, when incongruent (see Horner and Henson 2009; Valt et al. 2015).

In the present study, two different priming conditions for congruent and incongruent responses were obtained by presenting pictures of celebrities in two separate study phases with different yes–no questions about their nationality (“German?” or “American?”). In a single test phase, all celebrities were presented again and participants had to answer one of the two previous questions; hence, half of the repeated celebrities were tested with the same question as in the study phase (congruent), the other half with the other question (incongruent). According to the congruency between the responses made at study and the response required at test, celebrities in one set were primed with the correct decision/action codes and stimuli in the other set were primed with the incorrect decision/action codes. Changing the question within the same semantic task substantially affects decision and action binding but spares classification binding. This procedure (study–study-test) was performed twice in two consecutive blocks, with the order of the questions in the study phases counterbalanced to control for potential order effects.

The present study investigated, firstly, the time course of automatic retrieval of the previously established decision/action codes, and, secondly, the effect of this retrieval on the preparation of the task-relevant response. Given its sensitivity to motor cortex activity, the LRP represents a promising method to investigate the retrieval of action/decision codes and the subsequent effect on the preparation of the task-relevant response. Retrieval of decision/action codes should result in a small deflection of LRP activity, with positive polarity for incongruent trials and negative polarity for congruent trials. Importantly, according to a dual-route framework, the activation of a task-irrelevant response by the direct route is fast and automatic because the response is triggered as soon as sufficient distinctive features are processed to activate the associated response codes, hence this deflection should occur early (i.e. before the main LRP deflection). On the other hand, the preparation of a task-relevant response is intentional and requires the complete re-processing of the stimulus according to the current task requirement. Therefore, the retrieval of response codes by the direct route should occur after early stages of stimulus processing, indexed by early visual potentials such as the N170 (Bentin et al. 1996), and before the onset of the main negative deflection signalling the preparation of the task-relevant response by the indirect route resulting from the conclusion of stimulus processing. In addition, as in cognitive conflict tasks, the retrieval of response codes bound to a stimulus might affect the current response selection processes, slowing the onset of the main negative deflection in incongruent as compared to congruent trials. Previous research on negative priming showed that the onset of the LRP main negative deflection is delayed when the response associated with a stimulus at study is different from the response required to the stimulus at test (Gibbons and Stahl 2008). Therefore, differences in onset times between primed trials in the present experiment are expected, with later onsets for incongruent than congruent trials.

## Methods

### Participants

Twenty-five German university students participated in this experiment for course credits or a €16 or £15 payment in Berlin (*N* = 17) and Bangor (*N* = 8), respectively. The data of one participant was discarded because overall accuracy was below 60%. Mean age of the remaining 24 participants (18 women) was 24 years (range 18–42). All participants had normal or corrected-to-normal vision and, according to the Edinburgh Handedness Inventory (Oldfield 1971), were right-handed except for one ambidextrous and one left-handed participant. The study was approved according to the Declaration of Helsinki by the ethics committee at the School of Psychology, Bangor University, and all participants gave written informed consent.

### Stimuli

A total of 300 black and white portraits of celebrities (mostly actors, singers, politicians, athletes and TV presenters) were used as stimuli. Half of the selected celebrities were of German nationality (90 men, 60 women) and the other half were Americans (90 men, 60 women).

An additional set of 24 pictures of celebrities, resembling the characteristics of the experimental stimulus set, was used in practice runs.

### Procedure

After receiving instructions, participants performed practice runs with 12 stimuli for each task. The main experiment consisted of two blocks, each having two study phases followed by a distracter task and one test phase. In each block, the two opposite questions (“Is the celebrity German?” or “Is the celebrity American?”) were presented separately in the two study phases, with their order being counterbalanced between blocks. An object-orientation task was performed as distracter task before each test phase. In both blocks, the same task was presented in the test phases. Participants had to make a yes/no judgement by button presses with the left and right index finger; instructions emphasised both speed and accuracy. The task at test, response buttons’ mapping and order of the tasks in the study phases were counterbalanced across participants.

In each study phase, 50 randomly selected stimuli (15 German males, 10 German females, 15 American males, and 10 American females) were presented three times. During the study phases stimuli were presented three times in semi-random order: a stimulus could not be shown for the second time before all stimuli had been presented once, and the third presentation started only when all stimuli had been presented twice; immediate repetitions were excluded. Stimuli from the study phases were presented again in the test phase (primed) mixed with 50 stimuli not previously shown (unprimed) in random order. According to the task at study, the response for primed stimuli presented in the test phase was either congruent (identical decision/action) or incongruent (reversed decision/action) to the response expressed at study. Between the study phases and the test phase a distracter phase of 70 stimuli (35 objects in their canonical position, 35 upside-down rotated objects, presented in random order) in an orientation judgement task was performed, which was not analysed.

Throughout the experiment, each stimulus was presented for 600 ms, followed by a fixation cross, displayed for 2400 ms in study and distracter phases and for 1900 ms in test phases. Responses were recorded during the whole duration of the trial (stimulus and fixation cross presentation). Portraits had a size of 8.6° × 11.6° (width × height) and were displayed on a black background.

In order to ensure priming effects, participants were always requested to be as fast and accurate as possible in responding to all the stimuli.

### EEG recording

The EEG was recorded from 27 Ag/AgCl electrodes embedded in an elastic cap. The locations of the electrodes were based on the International 10–20 system and corresponded to the positions: Fp1/2, F7/8, F3/4, Fz, FC3/4, FCz, T7/8, C3/4, Cz, CP3/4, CPz, P7/8, P3/4, Pz, PO9/10, O1/2. Two electrodes were applied directly to the skin or into the cap over the left and right mastoids, M1 and M2. Due to different EEG recording procedures in the two labs, M1 served as the initial common reference and AFz as ground in Berlin, and FCz served as initial common reference and Fpz as ground in Bangor. EOG was recorded from four electrodes, with two placed below the right and the left eye, measured against Fp1 and Fp2 (VEOG), and two on the outer canthi of both eyes (HEOG). All signals were digitised with a frequency of 250 Hz and band-pass filtered from 0.05 to 70 Hz. Electrode impedance was kept below 10 kΩ for EEG electrodes and below 20 kΩ for EOG electrodes.

### Data processing and analyses

Only data from the test phase are reported. Trials with early (RT < 200 ms), missing or incorrect responses were removed from the RT analysis. In addition, trials with primed stimuli were removed when one or more incorrect responses were given at study.

Offline, the influence of blinks and eye movements on the EEG signal was corrected via independent component analysis based on 20 calibration trials for each type of ocular artefact (left, right, up, and down movement, blink) obtained after the experiment proper. A low-pass filter with a cut-off frequency of 18 Hz was applied (roll off 48 dB/octave). The EEG was segmented into epochs anchored to the stimulus; epoch duration was 1100 ms, starting 100 ms before stimulus onset. Epochs for primed stimuli were selected similarly to the RT analysis, automatically and visually inspected to detect artefacts and, if present, discarded. The 100-ms pre-stimulus interval was used as baseline and all signals were re-calculated to average reference. Epochs were averaged according to task at study, resulting in two priming conditions (primed congruent and primed incongruent).

The LRP was calculated based on brain signals recorded at the electrode positions C3 and C4. Activity ipsilateral to the responding hand was subtracted from contralateral activity. The LRPs for right- and left-hand responses were then averaged separately according to priming condition. Two sets of analysis were performed to explore automatic retrieval of action/decision codes and subsequent effects on response preparation. Early automatic activations of response codes are generally evident as small dips preceding the main negative deflection, whereas response preparation is mostly evident as main LRP negative deflection.

To analyse the automatic retrieval of action/decision codes, mean amplitudes were calculated in four steps of 50-ms time-windows for the two priming conditions. This procedure was performed starting at 150 ms, when high-level perceptual analysis in the cortex begins, as indexed by early visual potentials, up to the onset-time of the main LRP deflection at 350 ms, indicating preparation of the task-relevant response. The activity in the obtained time-windows was first analysed with repeated-measures ANOVA including the factors time-window and congruency. Two-tailed paired-samples *t* tests were then calculated, and the significance level of *α* = 0.05 was adjusted according to Bonferroni’s correction for multiple pairwise tests (*α*/4 = 0.0125).

To determine the onset time of the main negative LRP deflection, a linear regression procedure was used based on jackknife averages (Miller et al. 1998). The onset time of the LRP was the point at which the regression line crosses the x-axis. An ANOVA with the factor priming (congruent vs. incongruent) was performed on the onset time of the jackknife averages. The *F* value was corrected according to Ulrich and Miller (2001), *F*_c_ = *F*/(*n* − 1)^2^.

## Results

The order of the tasks in the study phases did not significantly interact with the effect of response congruency in accuracy or in response times (RTs), *F*s < 1. Therefore, data were collapsed over blocks.

**Fig. 1:**
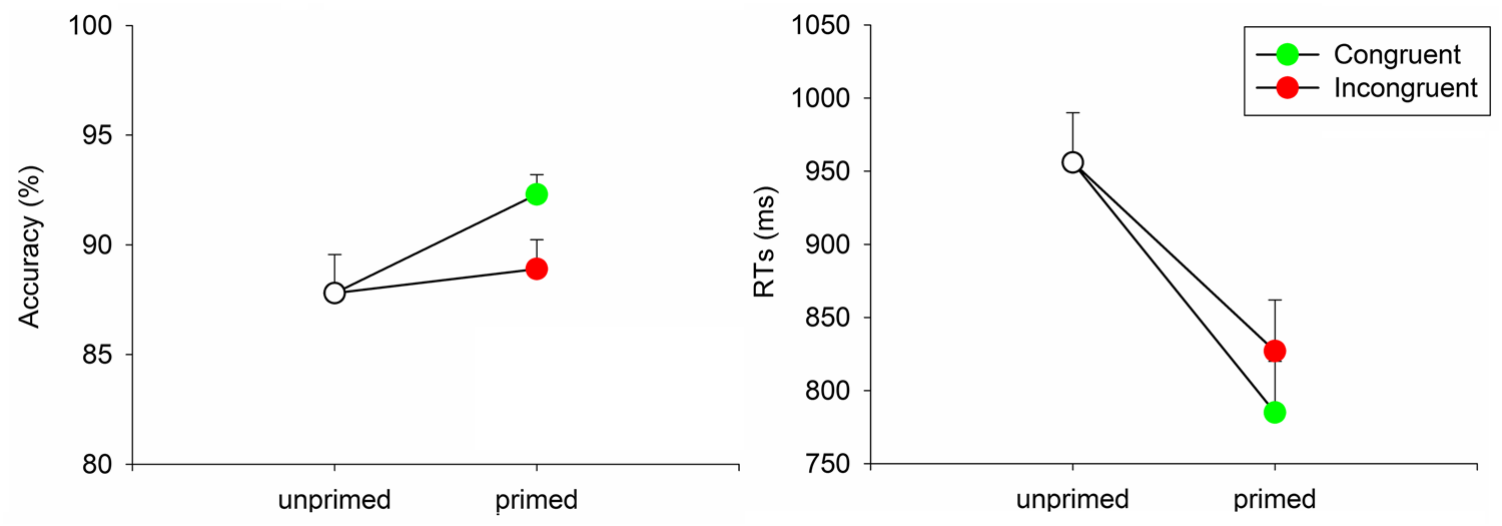
Mean accuracy and mean response times as a function of priming condition. *Error bars* represent the standard error (in the positive direction only)

Missing and too early responses (RT < 200 ms) accounted for 0.7% of the trials. Stimulus repetition significantly improved accuracy only when responses in the study and test phase were congruent, *t*(23) = 3.44, *p* = 0.002, but not when responses were incongruent, *t* < 1. The larger accuracy gain of 3.5% (SE = 1.1%) for congruent than incongruent responses was significant, *t*(23) = 3.16, *p* = 0.004 (see Fig. 1).

Further 16.2% of correct primed trials were discarded because of at least one incorrect response at study. Priming, assessed as difference between mean RTs for unprimed and primed stimuli, was significant for congruent responses, *t*(23) = 11.80, *p* < 0.001, and for incongruent responses, *t*(23) = 8.56, *p* < 0.001. The response congruency effect, assessed as the difference between the two priming conditions (*M* = 42 ms, SE = 9), was significant, *t*(23) = 4.48, *p* < 0.001 (see Fig. 1).

**Fig. 2:**
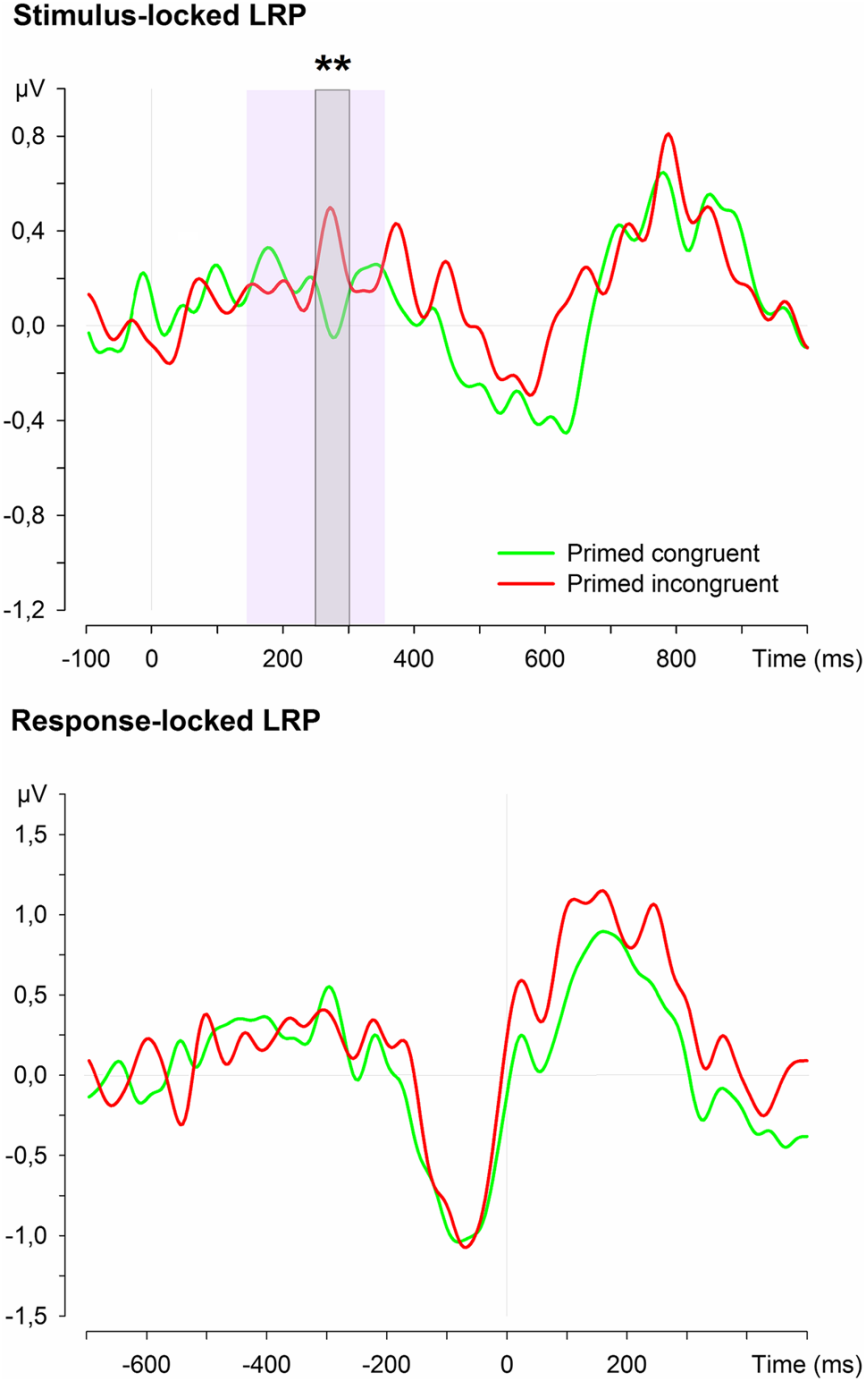
Stimulus-locked and response-locked grand average LRPs for primed stimuli bound to a congruent or an incongruent response. The *light grey background* highlights the analysed interval; *dark grey* marks the significant time-window

Average LRP activity for primed congruent and incongruent stimuli was calculated for the time-windows 150–200, 200–250, 250–300, and 300–350 ms. The ANOVA with congruency and time-window as factors showed no main effects of time-window and congruency, *F* < 1, but a significant interaction between these factors, *F*(3,21) = 3.99, *p* = 0.021, η_p_^2^ = 0.363. Follow-up comparisons of the individual 50-ms time-windows of the LRP activity in the two congruency conditions, with adjusted α levels of 0.0125, showed a single significant difference in the time-window 250–300 ms, *t*(23) = 2.81, *p* = 0.010, *d* = 0.57 (see Fig. 2). Confirming the hypothesis, the LRP for primed incongruent showed a positive deflection while the LRP for primed congruent presented a negative deflection. No other time-windows did present any significant differences, *t*s(23) < 1.01, *p*s > 0.323.

Although the latencies of the main negative deflections were numerically shorter for primed congruent (*M* = 355 ms) than primed incongruent (*M* = 373 ms), the difference in onset times was not significant, *F*_c_(1,23) = 1.35, *p*_c_ = 0.257.

## Discussion

According to the theory of rapid response learning, priming can result from the retrieval of stimulus–response bindings created during previous presentations of a stimulus. Here, the timing of the retrieval of decision/action codes was tested in a repetition priming experiment, where the task at test was identical/reversed to the task at study. As predicted by rapid response learning, reversal of the question at test produced a significant decrease in accuracy and significantly reduced response speed for celebrities primed with incongruent decision/action codes compared to celebrities primed with congruent decision/action codes. However, response speed was still faster for primed than unprimed stimuli, irrespective of decision/action congruency, indicating the presence of priming resistant to changes in response codes. Residual priming for primed incongruent trials is evidence of facilitation in perceptual and conceptual networks, as reported in a previous investigation on repetition priming for faces (Valt et al. 2015).

Referring to the electrophysiological activity associated with the retrieval of decision/action codes, when the response bound to a stimulus at study was congruent to the response at test, the LRP presented a small negative deflection in the 250–300 ms time-window. In contrast, the LRP in this time-window showed a positive deflection when responses at study and test were incongruent. A negative deflection of the LRP indicates the activation of the correct hand and a positive deflection reflects activation of the incorrect hand. These LRP results showed that the previously bound decision and action codes influence motor cortex activity at around 250 ms, regardless of their adequacy to the new task requirements. This motor cortex activity provides direct support to the predictions that retrieved response codes bound to a stimulus are automatically transmitted to the motor cortex when the stimulus is repeated. Additionally, these results move substantially forward the time when these bindings are retrieved, as suggested by previous electrophysiological studies (at around 450 ms in Race et al. 2010). However, the absence of a significant difference in the onset latency of the main negative deflection does not substantiate a delay of response preparation by incongruent decision/action codes.

The present results show that stimulus–decision and stimulus–action bindings are established and are retrieved early during stimulus processing. Race et al. (2010) reported the only previous evidence of decision/action code retrieval locked to the onset of the stimulus at around 450 ms after stimulus onset; that is 150 ms after the effect observed in the present experiment. This late effect could be dependent on specific aspects of the employed design: for example, the required semantic judgement was presented only half a second before each stimulus, and not at the beginning of the phase as in the present experiment, or because written words were used instead of pictures of celebrities. Our results do not corroborate the response-locked ERP effect related to the joint effect of classification, decision, and action codes observed by Horner and Henson (2012) (see also Race et al. 2010). In fact, the present experiment shows that the retrieval of decision/action codes is time-locked to stimulus processing (see Fig. 2 for the response-locked LRP), although the classification code was not manipulated here. Therefore, the response-locked effect observed by Horner and Henson (2012) might reflect differences in code retrieval when the task manipulation influences also the classification code. The lack of any evidence of ERP and behavioural effects of action code retrieval in Hsu and Waszak (2012) could instead be related to the fact that stimuli were primed only by a single presentation at study and, therefore, no strong stimulus–action binding may have been established (Schnyer et al. 2007; Valt et al. 2015).

The timing of the present LRP effect related to decision/action code retrieval matches well with many other ERP studies of short- and long-lived memory for faces. Paller et al. (2003) and Boehm et al. (2006) reported long-lived data driven priming for faces starting at 270–300 ms after stimulus onset. Around 250 ms there is also a highly consistent ERP component for short-lived memory. The so-called ERE/N250r (Schweinberger et al. 1995) is an ERP component associated with perceptual identification of a face, potentially related to the activity of the fusiform cortex (Schweinberger et al. 2004). This component seems to reflect activity in the Face Recognition Unit (FRU) of Bruce and Young´s model (Bruce and Young 1986, 2012), a stage in which a facial stimulus is recognised as a familiar face. These studies provide converging evidence that after about 250 ms a face is analysed sufficiently to enable memory processes that depend on processing of the identity of the face to occur.

As reviewed above, stimuli are bound to three response codes by rapid response learning: classification, decision, and action (Horner and Henson 2009). The present design, by changing the response within the same semantic task, did not impact the classification code: participants classify German celebrities as German, both when asked “is the celebrity German?” or when asked “is the celebrity American?”. On the other hand, changing the question within the same semantic task affects the decision code (e.g. from yes to no) and the action code (e.g. from right to left button press). It follows that the observed LRP activity might reflect the concurrent retrieval of both the action and the decision codes. Previous studies showed that these two codes can be isolated by changing the mapping of the response buttons within the same question, affecting only the action code, or when the question is different, impacting only the decision code (Dennis and Perfect 2013; Hsu and Waszak 2012). Future studies should consider these experimental manipulations to further investigate the electrophysiological activity associated with the separate retrieval of action and decision codes.

In conclusion, the observed early activation of decision/action codes is direct evidence that a response bound to a primed stimulus is retrieved and transmitted to the motor cortex, as suggested by the rapid response learning theory (Dobbins et al. 2004) and other instance theories (Hommel 2004; Logan 1990). In addition, the early onset time of action/decision retrieval fits well the assumption made by dual-route models that task-irrelevant responses are retrieved at early stages of stimulus processing when sufficient stimulus features are processed. A dual-route theory for priming, like the multiple-routes multiple-code framework suggested by Horner and Henson (2009) and by Valt et al. (2015), finds clear support from the present findings. It follows that Burton’s (1998) model for repetition priming in person recognition, and models for object recognition (Humphreys et al. 1995), could be extended in order to fully account for repetition priming with a route where response codes can be pre-activated and with a response stage where different responses interact (Valt et al. 2015). Moreover, the present experiment shows that the LRP can be used as a potential tool to further analyse the time course of stimulus processing.
